# The effect of *CYP7B1* polymorphisms on the risk of coronary heart disease in Hainan Han population

**DOI:** 10.1186/s12920-021-01067-x

**Published:** 2021-09-07

**Authors:** Tiebiao Liang, Xianbo Zhang, Anshan Liang, Haiqing Wu, Qi Wang, Jun He, Ming Long, Tianbo Jin

**Affiliations:** 1 Department of Cardiovascular Internal Medicine, People’s Hospital of Wanning, Wanning, 571500 Hainan China; 2 Department of General Practice, Affiliated Haikou Hospital of Xiangya Medical College, Haikou, 570311 Hainan China; 3 Key Laboratory of Molecular Mechanism and Intervention Research for Plateau Diseases of Tibet Autonomous Region, School of Medicine, Xizang Minzu University, Xianyang, 712082 Shaanxi China; 4 Key Laboratory of Resource Biology and Biotechnology in Western China, Ministry of Education, Northwest University, Xi’an, 710069 Shaanxi China

**Keywords:** Coronary heart disease, *CYP7B1* polymorphisms, Susceptibility

## Abstract

**Background:**

Coronary heart disease (CHD) is the leading cause of human death worldwide. Genetic factors play an important role in the occurrence of CHD. Our study is designed to investigate the influence of *CYP7B1* polymorphisms on CHD risk.

**Methods:**

In this case–control study, 508 CHD patients and 510 healthy individuals were recruited to determine the correlation between *CYP7B1* polymorphisms (rs7836768, rs6472155, and rs2980003) and CHD risk. The associations were evaluated by computing odds ratios (OR) and 95% confidence intervals (CI) with logistic regression analysis. The association between SNP-SNP interaction and CHD susceptibility was carried out by multifactor dimensionality reduction analyses.

**Results:**

Our study found that rs6472155 is significantly associated with an increased risk of CHD in age > 60 years (OR 2.20, 95% CI = 1.07–4.49, *p* = 0.031), women (OR 3.17, 95% CI = 1.19–8.44, *p* = 0.021), and non-smokers (3.43, 95% CI = 1.16–10.09, *p* = 0.025). Rs2980003 polymorphism has a lower risk of CHD in drinkers (OR 0.47, 95% CI = 0.24–0.91, *p* = 0.025). Further analyses based on false-positive report probability validated these significant results. Besides, it was found that rs6472155 polymorphism was associated with uric acid level (*p* = 0.034).

**Conclusion:**

Our study indicated that *CYP7B1* polymorphisms are related to the risk of CHD, which provides a new perspective for prevent of CHD.

**Supplementary Information:**

The online version contains supplementary material available at 10.1186/s12920-021-01067-x.

## Introduction

Coronary heart disease (CHD) is a kind of heart disease caused by myocardial ischemia, hypoxia or necrosis caused by structural and functional changes of coronary artery, also known as atherosclerotic heart disease, coronary disease or ischemic heart disease. It is the most common type of cardiovascular disease in the world [1–3]. In America, about 620,000 people develop new coronary artery disease every year, and about 295,000 people have recurrent attacks [4]. The incidence and mortality of CHD are increasing year by year in China, and the incidence population is also getting younger and younger. Deaths caused by CHD rank the second among deaths caused by other diseases [5]. The World Health Organization (WHO) predicts that the number of CHD deaths will increase to 23.3 million by 2030, becoming the leading cause of human death [6]. The pathophysiological basis of CHD is atherosclerosis caused by a variety of pathogenic factors, resulting in stenosis or complete occlusion of blood vessels, reduction, or complete interruption of coronary blood flow, and eventually ischemia or necrosis of cardiomyocytes [7]. Although the exact pathogenesis of coronary heart disease is not very clear, the most widely accepted view is that coronary heart disease is a polygenetic disease, which is the result of the interaction between multiple genes and environmental factors [8]. In addition to the classic risk factors such as age, smoking, drinking, hypertension, diabetes, and hypercholesterolemia [9], a large number of studies have confirmed that genetic factors play an important role in the occurrence and development of CHD [10]. Besides, many genetic variants such as *LPA* [11], *CYP24A1* [12], *CYP2C19* [13], *PNPLA3 I148M* [14], and *IL-7*/*7R* [15] can significantly affect the susceptibility of CHD.

Cytochrome P450 (CYP), a superfamily of cystine-heme enzymes, its central role in the pathogenesis, progression, and prognosis of CHD has been determined [16]. *CYP7B1* (Cytochrome P450 Family 7 Subfamily B Member 1) is the subfamily member of CYP enzymes, involved in the metabolism of endogenous hydroxysterols and steroids such as neurosteroids [17, 18]. The *CYP7B1* gene mainly in human liver, brain, and reproductive tract [18]. 27-hydroxycholesterol (27HC) is a rich cholesterol metabolite, which increases in hypercholesterolemia and is found to be a competitive estrogen receptor antagonist in vascular system in atherosclerotic lesions. Umetani et al. found that increasing 27HC levels in mice caused by genetic manipulation (by knockout of the *CYP7B1* gene) could decrease estrogen-dependent vascular nitric oxide synthase expression and inhibited carotid artery endothelialization [19]. This finding indicated that *CYP7B1* may play an important role in the occurrence of CHD. It is well accepted that genetics polymorphism can significantly influence the gene expression. However, it is not clear whether *CYP7B1* genetic polymorphism affects the risk of CHD.

Thus, we performed a case–control study to determine the potential role of *CYP7B1* genetic variants in CHD patients. We obtained the SNPs in the *CYP7B1* gene in accordance with 1000 Human Genomic Projects. We further investigated the association between the *CYP7B1* polymorphisms and CHD risk in the Chinese Han population. Stratified analyses were carried out to evaluate the association. We also detect the relationship of SNP-SNP interaction in CHD with clinical indicators among genotypes. This study will give new horizon for the molecular mechanism in the CHD.

## Materials and methods

### Study population

In this case–control study, we recruited 1018 unrelated Chinese individuals including 508 patients with CHD and 510 age/gender-matched healthy subjects at Affiliated Haikou Hospital of Xiangya Medical College. All participants were told the purpose of study and signed written informed consents. Patients were firstly diagnosed and confirmed to be CHD by experienced cardiologists in accordance with coronary angiography [20]. The patients with congenial and rheumatic heart disease, a family history of CHD, a history of any atherosclerotic vascular diseases and other comorbidity such as chronic renal failure, malignancy, and chronic infections should be excluded. The healthy controls were selected from the CHD-free participants who take a normal physical examination in the same hospital. And the controls must meet the following inclusion criterion: (1) without a family history of CHD; (2) no diabetes and hypertension; (3) no cardiovascular and cerebrovascular diseases. Basic characteristics included age, gender, smoking status, drinking status, diabetes and hypertension status were acquired by medical records and questionnaire survey. Our study has been approved by the Medical Ethics Committee of Affiliated Haikou Hospital of Xiangya Medical College. Experiments in this study were performed following the protocol of Helsinki’s Declaration.

### Selection and genotyping for SNP

In our study, rs7836768, rs62519827, rs62519841, rs10808739, rs13276608, rs6472155, and rs2980003 in the *CYP7B1* gene were obtained from 1000 Genomes Project database with a minor allele frequency (MAF) > 5% for further studying. Genomic DNA from peripheral blood samples of all participants was extracted following the protocol of DNA extraction kit (Xi’an GoldMag Co. Ltd., Xi’an, China) [21]. We further designed the primers for PCR amplification according to Agena Bioscience Assay Design software. All SNPs were genotyped by an Agena MassARRAY iPLEX platform (Agena Bioscience Inc., CA, USA) [22]. The PCR reaction consisted of 1 μL of 10 ng/μL genomic DNA and 4 μL of PCR mixture that contained 1.8 μL of water, 0.5 μL of 10 × PCR buffer, 0.4 μL of 25 mM MgCl_2_, 0.1 μL of 25 mM dNTP, 1 μL of PCR Primer mix and 0.2 μL of 5 U/μL PCR Taq. The PCR conditions were as follows: initial denaturing at 95 °C for 2 min, followed by 45 cycles of denaturing at 95 °C for 30 s, annealing at 56 °C for 30 s, and final extension at 72 °C for 60 s. Then the final step is to keep it at 25 °C indefinitely. Matrix-assisted laser desorption/ionization-time of flight (MALDI-TOF) mass spectrometry was used to identify SNP alleles of different quality extension primers after alkaline phosphatase reaction, single group extension and resin desalination reaction [23]. Finally, the data of SNP genotyping was management and analyzed by Agena Bioscience TYPER 4.0 software [24].

### Bioinformatics analysis

Online software for HaploReg v4.1 (https://pubs.broadinstitute.org/mammals/haploreg/haploreg.php) was used to predict the possible functional effects on these SNPs.

### Statistical analysis

We performed SPSS version 17.0 software for statistical analyses. The *p*-value with two-tailed test lower than 0.05 means statistically significant. Data distributions are firstly evaluated for normality using the Kolmogorov–Smirnov test. Differences of age and clinical indicators between cases and controls were respectively tested by the student’s *t*-test and Mann–Whitney test. And the comparisons of gender between the cases and controls was compared by Pearson′s *χ*^*2*^ test. The Hardy–Weinberg equilibrium (HWE) for SNPs in the controls was evaluated by a Chi-squared test. The *χ*^*2*^ test or exact test was used to analyze the allele and genotype distributions of SNPs in the case and control groups. Association of *CYP7B1* polymorphisms with CHD susceptibility was determined by computing ORs and 95% Cis using logistic regression analysis under five multiple genetic models. The explanation for the genetic models is as following: If *A* is the wild-type allele, *B* is the mutant allele, *AA* is the wild homozygous genotype, *AB* is the mutant heterozygous genotype, and *BB* is the mutant homozygous genotype, then the five genetic models are defined as follows: (1) codominant model: *BB* Vs. *AA*, *AB* Vs. *AA* (*AA* was the reference); (2) dominant model: (*AB* + *BB*) Vs. *AA* (*AA* was the references); (3) recessive model: *BB* Vs. (*AA* + *AB*) (*AA* + *AB* was the reference); (4) log-additive model: *AA* Vs. *AB* Vs. *BB*; (5) allele model: *B* Vs. *A* (*A* was the reference). Besides, we also detected the associations stratified by age, gender, smoking, and drinking status. The false-positive report probability (FPRP) analysis was performed to validate these significant results in this study [25]. In the end, we explored the relationship of SNP interaction and CHD susceptibility with multifaceted dimensionality reduction (MDR) method in which the interaction model with the highest cross-validation consistency (CVC) and testing accuracy was considered best. The comparisons between clinical indicators and SNPs were detected by ANOVA test and one-way analysis.

## Results

### Study subjects

Our study included in 508 CHD patients and 510 healthy individuals. The basic information of the cases and controls were shown in Table 1. The average age was 62.17 ± 10.34 years in the cases and 61.12 ± 9.02 years in the controls. There was no significant differences in age, gender, and urea between the cases and controls (*p* = 0.084, *p* = 0.964, *p* = 0.759, respectively). There were significant comparisons in creatinine, uric acid, total-cholesterol, and apolipoprotein AI between the cases and controls (all *p* < 0.05).

**Table 1:** Basic characteristics of CHD patients and controls

Characteristics	Cases (n = 508)	Controls (n = 510)	*p*
*Age, years (mean* ± *SD)*^*a*^	62.17 ± 10.34	61.12 ± 9.02	0.084
> 60	282 (55.5%)	284 (55.7%)	
≤ 60	226 (45.5%)	226 (44.3%)	
*Gender *^*b*^			0.964
Male	334 (65.7%)	336 (65.9%)	
Female	174 (34.3%)	174 (34.1%)	
Urea (mmol/l)^c^	462.01 ± 4.50	456.63 ± 2.67	0.759
Creatinine (umol/l)^c^	384.73 ± 3.40	456.07 ± 3.50	< 0.001
Uric acid (umol/l)^c^	431.26 ± 1.08	489.10 ± 2.07	< 0.001
Total-cholesterol (mmol/l)^c^	369.60 ± 1.00	548.04 ± 1.05	< 0.001
Apolipoprotein AI (g/l)^c^	247.13 ± 5.74	351.08 ± 0.70	< 0.001
*Smoking status*			
Smoker	231 (45.5%)	115 (22.5%)	
Nonsmoker	186 (36.6%)	167 (32.7%)	
Missing	91 (17.9%)	228 (44.8%)	
*Drinking status*			
Drinking	52 (10.2%)	124 (24.3%)	
Nondrinking	306 (60.2%)	135 (26.5%)	
Missing	150 (29.6%)	251 (49.2%)	
Diabetes	190 (37.4%)		
Non diabetes	318 (62.6%)		
Hypertension	362 (71.3%)		
Normal tension	146 (28.7%)		

*p*^a^ value was calculated by Student ′s *t*-test. *p*^b^ value was calculated by Pearson ′s χ^2^ test

*p*^c^ Mann–whitney test is used

### Association analyses between *CYP7B1* polymorphisms and CHD risk

A total of seven SNPs (rs7836768, rs62519827, rs62519841, rs10808739, rs13276608, rs6472155, and rs2980003) in the *CYP7B1* gene were successfully detected in our study. The information of each SNP was presented in Table 2. The MAF for rs62519827, rs62519841, rs10808739, and rs13276608 were lower than 0.05, these polymorphisms were deleted in the current study. SNPs including rs7836768, rs6472155, and rs2980003 in the control followed HWE (*p* > 0.05). We investigated the relationship of *CYP7B1* genetic variants and the risk of CHD under five genetic models, and our result showed that there are no significant associations (Table 3).

**Table 2:** Allele frequencies among *CYP7B1* SNPs

SNP ID	Chromosome position	Alleles (minor/major)	MAF		O (HET)	E (HET)	*p*-HWE	HaploReg v4.1
			Case	Control				
rs7836768	chr8: 64,474,910	*G*/*A*	0.413	0.396	0.514	0.478	0.115	Enhancer histone marks, DNAse, Motifs changed
rs62519827	chr8: 64,569,390	*C*/*T*	0.003	0.005	0.010	0.010	1.000	Siphy cons, Enhancer histone marks, DNAse, Motifs changed
rs62519841	chr8: 64,588,948	*A*/*G*	0.005	0.005	0.010	0.010	1.000	Motifs changed
rs10808739	chr8: 64,727,703	*A*/*G*	0.029	0.042	0.084	0.081	1.000	DNAse, NHGRI/EBI GWAS hits
rs13276608	chr8: 64,769,294	*T*/*C*	0.001	0.003	0.006	0.006	1.000	Motifs changed, Selected eQTL genes
rs6472155	chr8: 64,817,650	*G*/*A*	0.286	0.264	0.422	0.388	0.067	Promoter histone marks, Enhancer histone marks, DNAse, Proteins bound, Motifs changed, NHGRI/EBI GWAS hits, GRASP QTL hits, Selected eQTL genes
rs2980003	chr8: 65,087,728	*T*/*C*	0.357	0.346	0.452	0.452	1.000	DNAse, NHGRI/EBI GWAS hits

SNP, Single nucleotide polymorphisms; MAF, Minor allele frequency; HWE, Hardy–Weinberg equilibrium; O (HET), Observed heterozygosity; E (HET), Expected heterozygosity; OR, Odds ratio; 95% CI, 95% confidence intervals

*p*^a^ values were calculated by exact test

**Table 3:** Association of *CYP7B1* polymorphisms with CHD risk

SNP ID	Model	Allele/Genotype	Case N	Control N	OR (95% CI)	*p*
rs7836768	Allele	*A*	595	616	1	
		*G*	419	404	1.07 (0.90–1.28)	0.431
	Codominant	*AG*	239	71	0.90 (0.69–1.19)	0.464
		*GG*	90	262	1.27 (0.87–1.84)	0.220
		*AA*	178	177	1	
	Dominant	*AG*-*GG*	329	333	0.98 (0.76–1.27)	0.878
	Recessive	*AA*-*AG*	417	248	1	
		*GG*	90	262	1.34 (0.96–1.89)	0.090
	Log-additive	–	–	–	1.08 (0.90–1.29)	0.427
rs6472155	Allele	*A*	725	751	1	
		*G*	291	269	1.12 (0.92–1.36)	0.252
	Codominant	*AG*	219	215	1.08 (0.83–1.39)	0.575
		*GG*	36	27	1.39 (0.82–2.35)	0.226
		*AA*	253	268	1	
	Dominant	*AG*-*GG*	255	242	1.11 (0.87–1.42)	0.404
	Recessive	*AA*-*AG*	472	483	1	
		*GG*	36	27	1.34 (0.80–2.25)	0.265
	Log-additive	–	–	–	1.12 (0.92–1.38)	0.260
rs2980003	Allele	*C*	653	666	1	
		*T*	363	352	1.05 (0.88–1.26)	0.587
	Codominant	*TC*	233	230	1.05 (0.81–1.37)	0.722
		*TT*	65	61	1.09 (0.73–1.63)	0.663
		*CC*	210	218	1	
	Dominant	*TC*-*TT*	298	291	1.06 (0.82–1.36)	0.657
	Recessive	*CC*-*TC*	443	448	1	
		*TT*	65	61	1.07 (0.73–1.55)	0.738
	Log-additive	–	–	–	1.05 (0.87–1.26)	0.626

CI, confidence interval; OR, odds ratio; SNP: single nucleotide polymorphism; OR, Odds ratio, 95% CI; 95% confidence intervals

*p*‐values were calculated by unconditional logistic regression analysis with adjustment for age and gender

*p* < 0.05 indicates statistical significance

### The association analyses stratified by age and gender

We then determined the effect of *CYP7B1* polymorphisms on the risk of CHD stratified by age and gender. Table 4 showed the associations under age-based stratification. We found that rs6472155 is significantly associated with an increased risk of CHD in age > 60 years (allele model: *G* vs *A*, OR 1.43, 95% CI = 1.10–1.86, *p* = 0.008; co-dominant model: *GG* vs *AA*, OR 2.20, 95% CI = 1.07–4.49, *p* = 0.031; log-additive: OR 1.39, 95% CI = 1.06–1.83, *p* = 0.018). On gender stratification (Table 5), our data indicated that rs6472155 can increase the susceptibility to CHD in women under allele model (*G* vs *A*, OR 1.48, 95% CI = 1.06–2.07, *p* = 0.022), co-dominant model (*GG* vs *AA*, OR 3.17, 95% CI = 1.19–8.44, *p* = 0.021), and recessive model (*GG* vs *AA*-*AG*, OR 2.91, 95% CI = 1.12–7.58, *p* = 0.029).

**Table 4:** Association between *CYP7B1* polymorphisms and CHD risk stratified by age

SNP ID	Model	Genotype	Case	Control	OR (95% CI)	*p*	Case	Control	OR (95% CI)	*p*
			> 60 years				≤ 60 years			
rs7836768	Allele	*A*	326	342	1		269	274	1	
		*G*	238	226	1.11 (0.87–1.40)	0.410	181	178	1.04 (0.79–1.35)	0.796
	Codominant	*AG*	140	156	0.91 (0.89–1.32)	0.624	99	106	0.92 (0.61–1.39)	0.693
		*GG*	49	35	1.52 (0.89–2.58)	0.123	41	36	1.13 (0.66–1.93)	0.669
		*AA*	93	93	1		85	84	1	
	Dominant	*AG*-*GG*	189	191	1.02 (0.71–1.45)	0.920	140	142	0.97 (0.66–1.43)	0.890
	Recessive	*AA*-*AG*	233	249	1		184	190	1	
		*GG*	49	35	1.61 (1.00–2.59)	0.052	41	36	1.18 (0.72–1.93)	0.519
	Log-additive	–	–	–	1.15 (0.90–1.48)	0.266	–	–	1.03 (0.80–1.34)	0.806
rs6472155	Allele	*A*	388	431	1		337	320	1	
		*G*	176	137	**1.43 (1.10–1.86)**	**0.008**	115	132	0.83 (0.62–1.11)	0.205
	Codominant	*AG*	124	111	1.31 (0.92–1.86)	0.130	95	104	0.82 (0.56–1.19)	0.293
		*GG*	26	13	**2.20 (1.07–4.49)**	**0.031**	10	14	0.64 (0.27–1.50)	0.300
		*AA*	132	160	1		121	108	1	
	Dominant	*AG*-*GG*	150	124	1.40 (1.00–1.95)	0.048	105	118	0.79 (0.55–1.15)	0.222
	Recessive	*AA*-*AG*	256	271	1		216	212	1	
		*GG*	26	13	1.94 (0.97–3.90)	0.062	10	14	0.70 (0.30–1.61)	0.403
	Log-additive	–	–	–	**1.39 (1.06–1.83)**	**0.018**	–	–	0.81 (0.59–1.10)	0.180
rs2980003	Allele	*C*	350	369	1		303	297	1	
		*T*	214	197	1.15 (0.90–1.46)	0.273	149	155	0.94 (0.72–1.24)	0.673
	Codominant	*TC*	126	137	0.97 (0.68–1.39)	0.856	107	93	1.20 (0.81–1.77)	0.368
		*TT*	44	30	1.52 (0.89–2.61)	0.126	21	31	0.70 (0.38–1.31)	0.268
		*CC*	112	116	1		98	102	1	
	Dominant	*TC*-*TT*	170	167	1.07 (0.76–1.50)	0.708	128	124	1.08 (0.74–1.56)	0.705
	Recessive	*CC*-*TC*	238	253	1		205	195	1	
		*TT*	44	30	1.55 (0.94–2.57)	0.087	21	31	0.64 (0.36–1.16)	0.142
	Log-additive	–	–	–	1.15 (0.90–1.48)	0.261	–	–	0.94 (0.72–1.24)	0.574

Bold indicate that *P* < 0.05 means the data are statistically significant

OR, Odds ratio, 95% CI; 95% confidence intervals

*p* values were calculated by logistic regression adjusted by age and gender

*p* < 0.05 indicates statistical significance

**Table 5:** Association between *CYP7B1* polymorphisms and CHD risk stratified by gender

SNP ID	Model	Genotype	Case	Control	OR (95% CI)	*p*	Case	Control	OR (95% CI)	*p*
			Women				Men			
rs7836768	Allele	*A*	205	223	1		390	393	1	
		*G*	141	125	1.23 (0.90–1.67)	0.191	278	279	1.00 (0.81–1.25)	0.971
	Codominant	*AG*	89	95	1.00 (0.63–1.59)	0.989	150	167	/	/
		*GG*	26	15	1.87 (0.90–3.89)	0.096	64	56	1.08 (0.69–1.68)	0.734
		*AA*	58	64	1		120	113	1	
	Dominant	*AG*-*GG*	115	110	1.12 (0.72–1.75)	0.616	214	223	0.90 (0.66–1.24)	0.537
	Recessive	*AA*-*AG*	147	159	1		270	280	1	
		*GG*	26	15	1.86 (0.94–3.67)	0.073	64	56	1.19 (0.80–1.77)	0.392
	Log-additive	–	–	–	1.24 (0.89–1.73)	0.201	–	–	1.01 (0.81–1.25)	0.961
rs6472155	Allele	*A*	239	266	1		486	485	1	
		*G*	109	82	**1.48 (1.06–2.07)**	**0.022**	182	187	0.97 (0.76–1.23)	0.812
	Codominant	*AG*	73	70	1.21 (0.78–1.89)	0.393	146	145	/	/
		*GG*	18	6	**3.17 (1.19–8.44)**	**0.021**	18	21	1.37 (0.83–2.26)	0.652
		*AA*	83	98	1		170	170	1	
	Dominant	*AG*-*GG*	91	76	1.37 (0.89–2.10)	0.148	164	166	0.99 (0.73–1.34)	0.937
	Recessive	*AA*-*AG*	156	168	1		316	315	1	
		*GG*	18	6	**2.91 (1.12–7.58)**	**0.029**	18	21	0.86 (0.45–1.64)	0.637
	Log-additive	*–*	–	–	1.45 (1.02–2.06)	0.037	–	–	0.97 (0.75–1.25)	0.803
rs2980003	Allele	*C*	225	215	1		428	451	1	
		*T*	123	133	0.88 (0.65–1.20)	0.432	240	219	1.16 (0.92–1.45)	0.212
	Codominant	*TC*	81	81	0.92 (0.58–1.46)	0.732	152	149	/	/
		*TT*	21	26	0.73 (0.37–1.43)	0.362	44	35	1.37 (0.83–2.26)	0.220
		*CC*	72	67	1		138	151	1	
	Dominant	*TC*-*TT*	102	107	0.88 (0.57–1.35)	0.551	196	184	1.16 (0.86–1.58)	0.334
	Recessive	*CC*-*TC*	153	148	1		290	300	1	
		*TT*	21	26	0.76 (0.41–1.43)	0.398	44	35	1.29 (0.81–2.08)	0.286
	Log-additive	–	–	–	0.87 (0.64–1.19)	0.394	–	–	1.15 (0.92–1.45)	0.220

Bold indicate that *P* < 0.05 means the data are statistically significant

OR, Odds ratio, 95% CI; 95% confidence intervals

*p* values were calculated by logistic regression adjusted by age and gender

*p* < 0.05 indicates statistical significance

### *CYP7B1* polymorphisms related to CHD susceptibility under smoking and drinking subgroups

We also detected the associations stratified by smoking and drinking status. As was shown in Table 6, rs2980003 polymorphism has a lower risk of CHD in drinkers under allele model (*T* vs *C*, OR 0.57, 95% CI = 0.35–0.95, *p* = 0.031), co-dominant model (*TC* vs *CC*, OR 0.48, 95% CI = 0.23–0.97, *p* = 0.042), dominant model (*TC*-*TT* vs *CC*, OR 0.47, 95% CI = 0.24–0.91, *p* = 0.025), and log-additive model (OR 0.59, 95% CI = 0.36–0.98, *p* = 0.041). In non-drinkers, we observed that rs6472155 significantly increased a risk of CHD (co-dominant model: *GG* vs *AA*, OR 3.16, 95% CI = 1.05–9.48, *p* = 0.040; log-additive model: OR 3.43, 95% CI = 1.16–10.09, *p* = 0.025).

**Table 6:** *CYP7B1* polymorphisms related to CHD risk stratified by smoking and drinking status

SNP ID	Model	Genotype	Smoking		Non-smoking		Drinking		Non-drinking	
			OR (95% CI)	*p*	OR (95% CI)	*p*	OR (95% CI)	*p*	OR (95% CI)	*p*
rs7836768	Allele	*A*	1		1		1		1	
		*G*	0.89 (0.65–1.23)	0.485	1.19 (0.87–1.61)	0.275	1.07 (0.68–1.71)	0.763	1.25 (0.93–1.68)	0.139
	Codominant	*AG*	0.84 (0.50–1.40)	0.495	0.96 (0.60–1.54)	0.881	0.65 (0.31–1.36)	0.250	1.18 (0.75–1.85)	0.465
		*GG*	0.83 (0.45–1.56)	0.565	1.51 (0.76–2.99)	0.236	1.31 (0.54–3.16)	0.546	1.71 (0.88–3.29)	0.111
		*AA*	1		1		1		1	
	Dominant	*AG*-*GG*	0.84 (0.52–1.35)	0.461	1.07 (0.68–1.66)	0.781	0.81 (0.41–1.60)	0.550	1.29 (0.84–1.98)	0.247
	Recessive	*AA*-*AG*	1		1		1		1	
		*GG*	0.92 (0.53–1.60)	0.774	1.07 (0.68–1.66)	0.781	1.66 (0.75–3.66)	0.209	1.55 (0.85–2.84)	0.156
	Log-additive	–	0.90 (0.67–1.23)	0.522	1.16 (0.84–1.59)	0.370	1.07 (0.68–1.68)	0.769	1.27 (0.94–1.73)	0.119
rs6472155	Allele	*A*	1		1		1		1	
		*G*	0.83 (0.59–1.18)	0.294	1.27 (0.90–1.77)	0.171	0.99 (0.59–1.66)	0.961	1.15 (0.83–1.59)	0.387
	Codominant	*AG*	0.92 (0.57–1.47)	0.724	0.93 (0.59–1.44)	0.735	1.36 (0.70–2.64)	0.371	0.84 (0.56–1.29)	0.425
		*GG*	0.55 (0.23–1.31)	0.175	2.68 (0.93–7.71)	0.068	0.26 (0.03–2.21)	0.220	**3.16 (1.05–9.48)**	**0.040**
		*AA*	1		1		1		1	
	Dominant	*AG*-*GG*	0.85 (0.54–1.33)	0.481	1.05 (0.68–1.60)	0.841	1.18 (0.61–2.26)	0.625	0.98 (0.65–1.47)	0.907
	Recessive	*AA*-*AG*	1		1		1		1	
		*GG*	0.57 (0.25–1.32)	0.190	2.77 (0.98–7.83)	0.055	0.23 (0.03–1.18)	0.170	**3.43 (1.16–10.09)**	**0.025**
	Log-additive	–	0.82 (0.57–1.17)	0.268	1.19 (0.83–1.69)	0.346	0.95 (0.55–1.64)	0.863	1.16 (0.83–1.63)	0.376
rs2980003	Allele	*C*	1		1		1		1	
		*T*	0.97 (0.70–1.36)	0.879	1.04 (0.77–1.41)	0.795	**0.57 (0.35–0.95)**	**0.031**	0.96 (0.72–1.30)	0.810
	Codominant	*TC*	1.07 (0.66–1.72)	0.790	1.06 (0.67–1.69)	0.805	**0.48 (0.23–0.97)**	**0.042**	0.98 (0.63–1.53)	0.927
		*TT*	0.86 (0.41–1.79)	0.682	1.03 (0.54–1.96)	0.928	0.44 (0.15–1.32)	0.143	0.93 (0.49–1.53)	0.833
		*CC*	1		1		1		1	
	Dominant	*TC*-*TT*	1.02 (0.65–1.60)	0.933	1.05 (0.68–1.63)	0.818	**0.47 (0.24–0.91)**	**0.025**	0.97 (0.64–1.48)	0.882
	Recessive	*CC*-*TC*	1		1		1		1	
		*TT*	0.83 (0.41–1.68)	0.603	1.00 (0.55–1.81)	0.995	0.61 (0.21–1.75)	0.359	0.94 (0.52–1.71)	0.849
	Log-additive	–	0.97 (0.69–1.36)	0.857	1.03 (0.75–1.39)	0.875	**0.59 (0.36–0.98)**	**0.041**	0.97 (0.72–1.31)	0.840

Bold indicate that *P* < 0.05 means the data are statistically significant

OR, Odds ratio, 95% CI; 95% confidence intervals

*p* values were calculated by logistic regression adjusted by age and gender

*p* < 0.05 indicates statistical significance

### FPRP results

In order to check the positive findings, we conducted the FPRP analysis with setting the FPRP threshold as 0.2. As was presented in Additional file 1: Table S1, at the prior probability of 0.25, all the significant results for the association of rs6472155 and rs2980003 with CHD risk remained noteworthy (all FPRP < 0.2). These results indicated that the significant results made sense.

### Effect of SNP-SNP interactions on CHD susceptibility

**Fig. 1:**
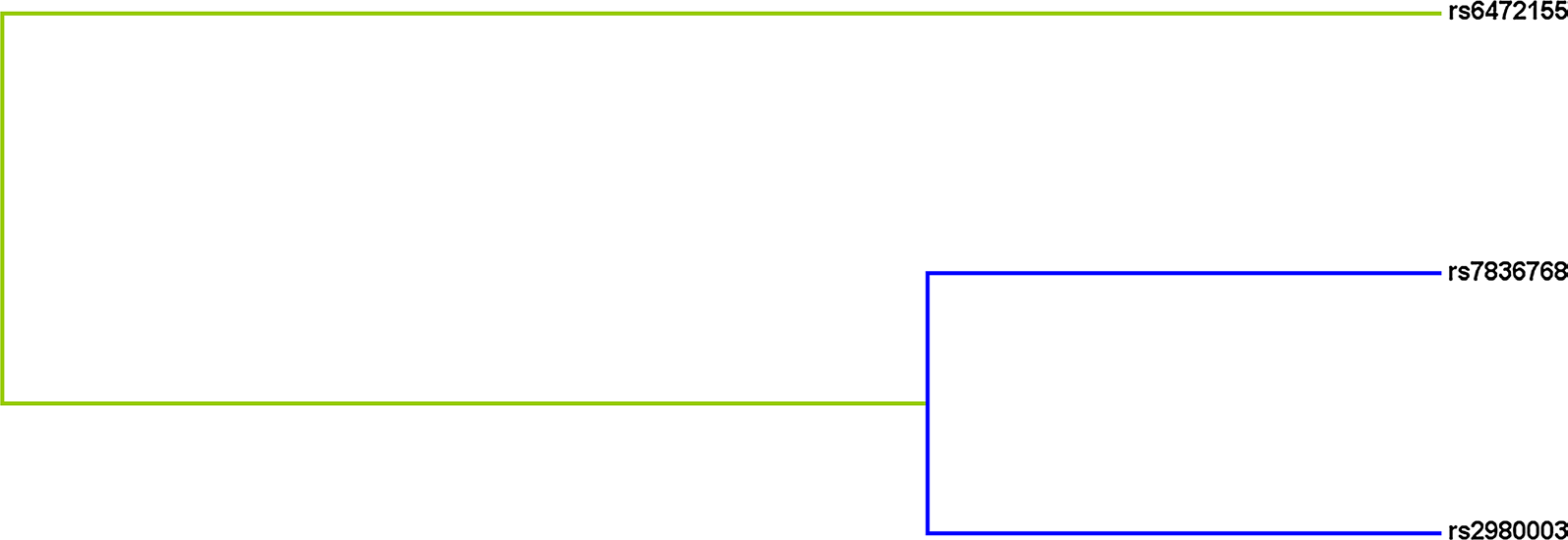
The tree diagram analysis among SNP interaction. The shorter the line connecting the 2 SNPs, the stronger the interaction. Green and blue line represent weak and no interactions

MDR analyses were performed to determine the influence of SNP-SNP interactions on the susceptibility to CHD. As were demonstrated in Table 7, the two-locus model included rs7836768 and rs6472155 (testing accuracy = 0.5079, CVC = 7/10, *p* = 0.021). The three-locus model was the combinations of rs7836768, rs6472155, and rs2980003 (testing accuracy = 0.4764, CVC = 10/10, *p* = 0.002). Thus, the best model for predicting CHD susceptibility was the three-locus model (OR 1.59, 95% CI = 1.19–2.13), which had the highest CVC. Additionally, the dendrogram showed weak or no interactions between the SNPs of the best models of CHD risk (Fig. 1).

**Table 7:** The analysis of SNP-SNP interaction models using MDR method

Model	Training Bal. Acc	Testing Bal. Acc	CVC	OR (95% CI)	*p*
rs7836768	0.5236	0.4931	8/10	1.19 (0.93–1.52)	0.167
rs7836768,rs6472155	0.5336	0.5079	7/10	**1.39 (1.05–1.83)**	**0.021**
rs7836768,rs6472155,rs2980003	0.5454	0.4764	10/10	**1.59 (1.19–2.13)**	**0.002**

Bold indicate that *P* < 0.05 means the data are statistically significant

Bal. Acc., Balanced accuracy; CVC, Cross-validation consistently

*p* values were calculated by χ^2^ test. *p* < 0.05 indicates statistical significance

### The association between SNP genotypes and clinical indicators

Finally, we studied the possible association of SNP with clinical indicators in CHD patients. As was showed in Table 8, the *AG* genotype (289.796 ± 84.427 umol/l) and *GG* genotype (296.096 ± 68.438 umol/l) of rs6472155 polymorphism were associated with a decreased uric acid concentration compared with the *AA* genotype (310.364 ± 86.950 umol/l) (*p* = 0.034).

**Table 8:** Comparisons between clinical characteristics and SNP genotypes

SNP	Urea (mmol/l)	Creatinine (umol/l)	Uric acid (umol/l)	Total-cholesterol (mmol/l)	Apolipoprotein AI (g/l)
rs7836768SNP					
*AA*	5.167 ± 1.422	2.350 ± 0.841	302.857 ± 82.083	4.024 ± 0.999	1.185 ± 0.225
*AG*	5.210 ± 1.592	2.370 ± 0.867	297.444 ± 87.826	4.066 ± 1.068	1.170 ± 0.238
*GG*	5.178 ± 1.556	2.430 ± 0.785	304.943 ± 84.697	4.186 ± 1.091	1.149 ± 0.255
*p*	0.958	0.761	0.718	0.501	0.518
rs6472155					
*AA*	5.209 ± 1.543	2.340 ± 0.825	310.364 ± 86.950	4.039 ± 1.083	1.179 ± 0.236
*AG*	5.160 ± 1.493	2.410 ± 0.888	289.796 ± 84.427	4.125 ± 1.011	1.162 ± 0.230
*GG*	5.186 ± 1.615	2.420 ± 0.753	296.096 ± 68.438	4.055 ± 1.045	1.165 ± 0.279
*p*	0.942	0.636	**0.034**	0.684	0.737
rs2980003					
*TT*	5.042 ± 1.387	2.300 ± 0.909	290.025 ± 86.984	3.885 ± 1.011	1.126 ± 0.223
*CT*	5.285 ± 1.599	2.370 ± 0.861	302.334 ± 81.741	4.075 ± 1.082	1.176 ± 0.224
*CC*	5.117 ± 1.474	2.410 ± 0.810	301.934 ± 88.426	4.142 ± 1.020	1.180 ± 0.254
*p*	0.384	0.639	0.569	0.239	0.261

*p* values were calculated by Kruskal–Wallis H test

Bold indicate that *P* < 0.05 means the data are statistically significan

## Discussion

In our study, we determined the influence of *CYP7B1* polymorphisms on CHD susceptibility. Our study showed that *CYP7B1* genetic variants significantly relate to the risk of CHD in Chinese Han population. Besides, we found that rs6472155 polymorphism was associated with uric acid level. To our best known, our study is the first time to investigate the effect of *CYP7B1* genetic variants on CHD susceptibility.

CHD is a kind of multifactorial disease resulting from environmental and genetic factor. An increasing number studies strongly supported that genetic polymorphisms involve in the risk of CHD, such as *SCARB1* [26], *RTEL1* [27], 5-*HTTLPR* [28], *CD40* [29], and *NT5C2* [30]. The *CYP7B1* gene is located on chromosome 8q21.3. *CYP7B1* is known to be involved in cholesterol synthesis of bile acids, which contributes to the CHD progression. Whereas there have been no studies on the genetic polymorphism in CHD. Therefore, we tried to evaluate the association between *CYP7B1* polymorphisms and CHD risk.

In our study, because the average age of case and control group is 60 years old, we stratified by age of 60 years. Our study found that rs6472155 polymorphism significantly affected the risk of CHD in age > 60 years but not in age ≤ 60 years, which demonstrated rs6472155 polymorphism as a risk factor of CHD in individuals aged 60 and older. Similar to our findings, Zhang et al. and Ye et al. reported that genetic polymorphism influence CHD risk in age > 60 years and age > 65 years [31, 32]. However, Huang et al. and Chen et al. showed that gene variants are associated with the susceptibility to CHD in age ≤ 61 years [30, 33]. Age is a risk factor for CHD. An epidemiologic study indicated that the incidence of CHD was eight to nine times greater in men and women who aged 55–64 years than in young patients [34]. The death rate due to CHD increased quickly in patients aged 55 years and was higher in patients aged 65 years or more than in young patients [35]. Previous studies have confirmed that genetic polymorphisms may play an important role in the pathogenesis of early onset CHD [36, 37]. These findings may suggest that the association of genetic variants with CHD risk relying on age and emphasize the importance of considering heterogeneity in genetic and CHD association study. We also noticed that rs6472155 polymorphism affected the susceptibility to CHD in women but not in men, which suggested that the impact of rs6472155 polymorphism on CHD risk presented gender difference. Our results seem to be consistent with other research which found genetic polymorphisms are associated with CHD risk in women [38]. In contrast, Luo et al., Ma et al., and Chen et al. indicated that genetic polymorphisms affect the risk of CHD in men [26, 30, 39]. Gender is also a risk factor for CHD. It has shown that men have higher cardiovascular disease morbidity and mortality rates than women, but the incidence of CHD increased significantly in postmenopausal women [40, 41]. These results revealed that innate differences in occurrence of CHD between women and men. It has been confirmed that gender differences could influence gene expression and then affect disease progression [42], and genetic polymorphism can impact the gene expression. Taken together, we guess that gender differences contribute to the occurrence of CHD depending on the polymorphism, further study was needed to confirm this hypothesis. Besides, rs6472155 polymorphism was related to CHD risk in non-drinkers but not in drinkers. It suggests that rs6472155 might play various roles in the development of CHD, and the risk association may depend on drinking.

Rs2980003 polymorphism has a lower risk of CHD in drinkers, whereas no relationship was found in non-drinkers, suggesting that rs6472155 may related to the risk of CHD relying on drinking status. Although there is no any studies focusing on the association of *CYP7B1* polymorphisms with cardiovascular disorders risk, we carried out the FPRP analysis to detect whether the positive findings in our study were just chance or noteworthy observations. The data showed that all of the significant findings remain noteworthy, which indicates our results make sense. In addition, the bioinformatics analysis revealed that rs6472155 might be affect the regulation of promoter histone marks, enhancer histone marks, DNAse, proteins bound, motifs changed, NHGRI/EBI GWAS hits, GRASP QTL hits, and selected eQTL genes, suggesting its possible functions in CHD. Rs2980003 might be associated with the regulation of DNAse and NHGRI/EBI GWAS hits. Several studies provided increasing evidence to support that SNPs confer susceptibilities by affecting gene expression [43–45]. Thus, we hypothesized that *CYP7B1* polymorphisms, especially rs6472155 and rs2980003 may affect the expression of *CYP7B1* to contribute to the risk of CHD. However, further study is necessary to confirm this hypothesis.

Given that SNP-SNP interactions are likely to be a ubiquitous component of the genetic architecture of common diseases [46]. The association of SNP-SNP interaction with CHD risk may help to discover the risk factors because of this disease caused by environmental and genetic interaction. We observed that the combinations of rs7836768, rs6472155, and rs2980003 are the best model to predict CHD.

Uric acid is the end-product of purine metabolism in humans, which play crucial roles in developing cardiovascular diseases including CHD [47]. Hyperuricemia is a potential risk factor for CHD [48, 49]. The possible mechanisms of uric acid induced CHD was that the elevated serum uric acid may lead to endothelial dysfunction through inflammation and oxidative stress and the formation of unstable lipid plaque in the coronary artery, which eventually leads to the occurrence of CHD [50]. Besides, it has been confirmed that serum uric acid can be used as prognostic marker of CHD [51]. Previous studies reveal that genetic polymorphisms can affect serum uric acid level. For example, the genetic polymorphisms in the *SAA1* gene was associated with serum uric acid levels, which have a high risk of hyperuricemia [52]. The *SLC2A9* rs11722228, *SF1* rs606458, and *GCKR* rs780094 variants modulate uric acid concentrations [53]. Moreover, Wang et al. showed that *APOE* polymorphism was associated with serum uric acid metabolism in patients with CHD [54]. Our data showed that patients with the rs6472155-*AA* genotype in the *CYP7B1* gene was associated with an increased uric acid level compared with the *AG* and *GG* genotype, indicating that carriers of the *A* allele of rs6472155 have a high risk of CHD. We assume that the *CYP7B1* allele may increase serum uric acid level and then exert its destructive endothelial dysfunction, which increases the risk of developing CHD. Of course, the above assumptions require more rigorous follow-up studies to confirm.

Some limitations exist in our present study. First, we investigated the association between *CYP7B1* polymorphisms and CHD susceptibility, the relationship between *CYP7B1* SNPs and the gene expression should be tested in future. Second, the molecular mechanism of *CYP7B1* in CHD is needed to be performed in the next work. In spite the above limitations, our study gives available information for the molecular mechanism of CHD in Chinese Han population.

## Conclusion

In summary, this study showed that rs6472155 is significantly related to an increased susceptibility to CHD in age > 60 years, women, and non-smokers. Rs2980003 polymorphism has a lower risk of CHD in drinkers. Besides, it was found that rs6472155 polymorphism was associated with uric acid level. These data may give a new potential biomarker for the prevention and management of CHD in Chinese Han population.

## Supplementary Information

**Additional file 1. Table S1** False-positive report probability analysis for the positive findings between CYP7B1 polymorphisms and CHD risk.

## Data Availability

The datasets generated during the current study are available in the figshare repository, https://doi.org/10.6084/m9.figshare.14915088.

## References

[1] Chen CY, Chuang SY, Fang CC, Huang LC, Hsieh IC, Pan WH, Yeh HI, Wu CC, Yin WH, Chen JW (2014). Gender disparities in optimal lipid control among patients with coronary artery disease. J Atheroscler Thromb.

[2] He Y, Zhang Z, Dai Q, Zhou Y, Yang Y, Yu W, An J, Jin L, Jerecic R, Yuan C, Li D (2012). Accuracy of MRI to identify the coronary artery plaque: a comparative study with intravascular ultrasound. J Magn Reson Imaging: JMRI.

[3] Sattelmair J, Pertman J, Ding EL, Kohl HW, Haskell W, Lee IM (2011). Dose response between physical activity and risk of coronary heart disease: a meta-analysis. Circulation.

[4] Mozaffarian D, Benjamin EJ, Go AS, Arnett DK, Blaha MJ, Cushman M, Das SR, de Ferranti S, Després JP, Fullerton HJ, Howard VJ, Huffman MD, Isasi CR, Jiménez MC, Judd SE, Kissela BM, Lichtman JH, Lisabeth LD, Liu S, Mackey RH, Magid DJ, McGuire DK, Mohler ER, Moy CS, Muntner P, Mussolino ME, Nasir K, Neumar RW, Nichol G, Palaniappan L, Pandey DK, Reeves MJ, Rodriguez CJ, Rosamond W, Sorlie PD, Stein J, Towfighi A, Turan TN, Virani SS, Woo D, Yeh RW, Turner MB (2016). Executive summary: heart disease and stroke statistics–2016 update: a report from the American Heart Association. Circulation.

[5] Joint Task Force for Guideline on the Assessment and Management of Cardiovascular Risk in China (2019). Guideline on the assessment and management of cardiovascular risk in China. Zhonghua yu fang yi xue za zhi [Chin J Prev Med].

[6] Hamrefors V (2017). Common genetic risk factors for coronary artery disease: new opportunities for prevention?. Clin Physiol Funct Imaging.

[7] Nakahara T, Dweck M, Narula N, Pisapia D, Narula J, Strauss H (2017). Coronary artery calcification: from mechanism to molecular imaging. JACC Cardiovasc Imaging.

[8] Deloukas P, Kanoni S, Willenborg C, Farrall M, Assimes T, Thompson J, Ingelsson E, Saleheen D, Erdmann J, Goldstein B, Stirrups K, König I, Cazier J, Johansson A, Hall A, Lee J, Willer C, Chambers J, Esko T, Folkersen L, Goel A, Grundberg E, Havulinna A, Ho W, Hopewell J, Eriksson N, Kleber M, Kristiansson K, Lundmark P, Lyytikäinen L, Rafelt S, Shungin D, Strawbridge R, Thorleifsson G, Tikkanen E, Van Zuydam N, Voight B, Waite L, Zhang W, Ziegler A, Absher D, Altshuler D, Balmforth A, Barroso I, Braund P, Burgdorf C, Claudi-Boehm S, Cox D, Dimitriou M, Do R, Doney A, El Mokhtari N, Eriksson P, Fischer K, Fontanillas P, Franco-Cereceda A, Gigante B, Groop L, Gustafsson S, Hager J, Hallmans G, Han B, Hunt S, Kang H, Illig T, Kessler T, Knowles J, Kolovou G, Kuusisto J, Langenberg C, Langford C, Leander K, Lokki M, Lundmark A, McCarthy M, Meisinger C, Melander O, Mihailov E, Maouche S, Morris A, Müller-Nurasyid M, Nikus K, Peden J, Rayner N, Rasheed A, Rosinger S, Rubin D, Rumpf M, Schäfer A, Sivananthan M, Song C, Stewart A, Tan S, Thorgeirsson G, van der Schoot C, Wagner P, Wells G, Wild P, Yang T, Amouyel P, Arveiler D, Basart H, Boehnke M, Boerwinkle E, Brambilla P, Cambien F, Cupples A, de Faire U, Dehghan A, Diemert P, Epstein S, Evans A, Ferrario M, Ferrières J, Gauguier D, Go A, Goodall A, Gudnason V, Hazen S, Holm H, Iribarren C, Jang Y, Kähönen M, Kee F, Kim H, Klopp N, Koenig W, Kratzer W, Kuulasmaa K, Laakso M, Laaksonen R, Lee J, Lind L, Ouwehand W, Parish S, Park J, Pedersen N, Peters A, Quertermous T, Rader D, Salomaa V, Schadt E, Shah S, Sinisalo J, Stark K, Stefansson K, Trégouët D, Virtamo J, Wallentin L, Wareham N, Zimmermann M, Nieminen M, Hengstenberg C, Sandhu M, Pastinen T, Syvänen A, Hovingh G, Dedoussis G, Franks P, Lehtimäki T, Metspalu A, Zalloua P, Siegbahn A, Schreiber S, Ripatti S, Blankenberg S, Perola M, Clarke R, Boehm B, O'Donnell C, Reilly M, März W, Collins R, Kathiresan S, Hamsten A, Kooner J, Thorsteinsdottir U, Danesh J, Palmer C, Roberts R, Watkins H, Schunkert H, Samani N (2013). Large-scale association analysis identifies new risk loci for coronary artery disease. Nat Genet.

[9] Dalen J, Alpert J, Goldberg R, Weinstein R (2014). The epidemic of the 20(th) century: coronary heart disease. Am J Med.

[10] Lettre G, Palmer C, Young T, Ejebe K, Allayee H, Benjamin E, Bennett F, Bowden D, Chakravarti A, Dreisbach A, Farlow D, Folsom A, Fornage M, Forrester T, Fox E, Haiman C, Hartiala J, Harris T, Hazen S, Heckbert S, Henderson B, Hirschhorn J, Keating B, Kritchevsky S, Larkin E, Li M, Rudock M, McKenzie C, Meigs J, Meng Y, Mosley T, Newman A, Newton-Cheh C, Paltoo D, Papanicolaou G, Patterson N, Post W, Psaty B, Qasim A, Qu L, Rader D, Redline S, Reilly M, Reiner A, Rich S, Rotter J, Liu Y, Shrader P, Siscovick D, Tang W, Taylor H, Tracy R, Vasan R, Waters K, Wilks R, Wilson J, Fabsitz R, Gabriel S, Kathiresan S, Boerwinkle E (2011). Genome-wide association study of coronary heart disease and its risk factors in 8,090 African Americans: the NHLBI CARe Project. PLoS Genet.

[11] Dong H, Cong H (2020). Correlations between lipoprotein(a) gene polymorphisms and calcific aortic valve disease and coronary heart disease in Han Chinese. J Int Med Res.

[12] Qian P, Cao X, Xu X, Duan M, Zhang Q, Huang G (2020). Contribution of CYP24A1 variants in coronary heart disease among the Chinese population. Lipids Health Dis.

[13] Akkaif MA, Daud NAA, Sha’aban A (2021). The role of genetic polymorphism and other factors on clopidogrel resistance (CR) in an Asian population with coronary heart disease (CHD). Molecules.

[14] Wu JT, Liu SS, Xie XJ, Liu Q, Xin YN (2020). Independent and joint correlation of PNPLA3 I148M and TM6SF2 E167K variants with the risk of coronary heart disease in patients with non-alcoholic fatty liver disease. Lipids Health Dis.

[15] Sun Y, Yan J, Zhang J, Wang A, Zou J, Gao C (2020). Contribution of IL-7/7R genetic polymorphisms in coronary heart disease in Chinese Han population. Int Immunopharmacol.

[16] Elbekai RH, El-Kadi AO (2006). Cytochrome P450 enzymes: central players in cardiovascular health and disease. Pharmacol Ther.

[17] Wu Z, Martin KO, Javitt NB, Chiang JY (1999). Structure and functions of human oxysterol 7alpha-hydroxylase cDNAs and gene CYP7B1. J Lipid Res.

[18] Yantsevich AV, Dichenko YV, Mackenzie F, Mukha DV, Baranovsky AV, Gilep AA, Usanov SA, Strushkevich NV (2014). Human steroid and oxysterol 7α-hydroxylase CYP7B1: substrate specificity, azole binding and misfolding of clinically relevant mutants. FEBS J.

[19] Umetani M, Domoto H, Gormley AK, Yuhanna IS, Cummins CL, Javitt NB, Korach KS, Shaul PW, Mangelsdorf DJ (2007). 27-Hydroxycholesterol is an endogenous SERM that inhibits the cardiovascular effects of estrogen. Nat Med.

[20] Zhang L, Yuan F, Liu P, Fei L, Huang Y, Xu L, Hao L, Qiu X, Le Y, Yang X, Xu W, Huang X, Ye M, Zhou J, Lian J, Duan S (2013). Association between PCSK9 and LDLR gene polymorphisms with coronary heart disease: case-control study and meta-analysis. Clin Biochem.

[21] Guo X, Song J (2019). Impact of ANXA5 polymorphisms on glioma risk and patient prognosis. J Neuro-oncol.

[22] Thomas RK, Baker AC, Debiasi RM, Winckler W, Laframboise T, Lin WM, Wang M, Feng W, Zander T, MacConaill L, Lee JC, Nicoletti R, Hatton C, Goyette M, Girard L, Majmudar K, Ziaugra L, Wong KK, Gabriel S, Beroukhim R, Peyton M, Barretina J, Dutt A, Emery C, Greulich H, Shah K, Sasaki H, Gazdar A, Minna J, Armstrong SA, Mellinghoff IK, Hodi FS, Dranoff G, Mischel PS, Cloughesy TF, Nelson SF, Liau LM, Mertz K, Rubin MA, Moch H, Loda M, Catalona W, Fletcher J, Signoretti S, Kaye F, Anderson KC, Demetri GD, Dummer R, Wagner S, Herlyn M, Sellers WR, Meyerson M, Garraway LA (2007). High-throughput oncogene mutation profiling in human cancer. Nat Genet.

[23] Li B, Hu C (2019). Associations among genetic variants and intracranial aneurysm in a Chinese population. Yonsei Med J.

[24] Gabriel S, Ziaugra L, Tabbaa D (2009). SNP genotyping using the Sequenom MassARRAY iPLEX platform. Curr Protoc Hum Genet.

[25] Deng Y, Zhou L, Yao J, Liu Y, Zheng Y, Yang S, Wu Y, Li N, Xu P, Lyu L, Zhang D, Lyu J, Dai Z (2020). Associations of lncRNA H19 polymorphisms at MicroRNA binding sites with glioma susceptibility and prognosis. Mol Therapy Nucleic Acids.

[26] Ma R, Zhu X, Yan B (2018). SCARB1 rs5888 gene polymorphisms in coronary heart disease: a systematic review and a meta-analysis. Gene.

[27] Lu S, Zhong J, Wu M, Huang K, Zhou Y, Zhong Z, Li Q, Zhou H (2019). Genetic analysis of the relation of telomere length-related gene (RTEL1) and coronary heart disease risk. Mol Genet Genomic Med.

[28] Zhang LJ, Zeng XT, Zhao MJ, He DF, Liu JY, Liu MY (2020). The important effect of 5-HTTLPR polymorphism on the risk of depression in patients with coronary heart disease: a meta-analysis. BMC Cardiovasc Disord.

[29] Sultan CS, Weitnauer M, Turinsky M, Kessler T, Brune M, Gleissner CA, Leuschner F, Wagner AH, Hecker M (2020). Functional association of a CD40 gene single-nucleotide polymorphism with the pathogenesis of coronary heart disease. Cardiovasc Res.

[30] Chen X, Zhang Z, Wang X, Chen Y, Wang C (2020). NT5C2 Gene polymorphisms and the risk of coronary heart disease. Public Health Genomics.

[31] Ye HD, Li YR, Hong QX, Zhou AN, Zhao QL, Xu LM, Xu MQ, Xu XT, Tang LL, Dai DJ, Jiang DJ, Huang Y, Wang DW, Duan SW (2015). Positive association between PPARD rs2016520 polymorphism and coronary heart disease in a Han Chinese population. Genet Mol Res: GMR.

[32] Zhang YY, Zhou X, Ji WJ, Liu T, Ma J, Zhang Y, Li YM (2019). Association between CYP2C19*2/*3 polymorphisms and coronary heart disease. Curr Med Sci.

[33] Huang K, Zhong J, Li Q, Zhang W, Chen Z, Zhou Y, Wu M, Zhong Z, Lu S, Zhang S (2019). Effects of CDKN2B-AS1 polymorphisms on the susceptibility to coronary heart disease. Mol Genet Genomic Med.

[34] Konnov MV, Dobordginidze LM, Deev AD, Gratsiansky NA (2016). Own and parental predictors of low blood level of high density lipoprotein cholesterol in offspring of persons with early coronary heart disease. Kardiologiia.

[35] Roger VL, Go AS, Lloyd-Jones DM, Adams RJ, Berry JD, Brown TM, Carnethon MR, Dai S, de Simone G, Ford ES, Fox CS, Fullerton HJ, Gillespie C, Greenlund KJ, Hailpern SM, Heit JA, Ho PM, Howard VJ, Kissela BM, Kittner SJ, Lackland DT, Lichtman JH, Lisabeth LD, Makuc DM, Marcus GM, Marelli A, Matchar DB, McDermott MM, Meigs JB, Moy CS, Mozaffarian D, Mussolino ME, Nichol G, Paynter NP, Rosamond WD, Sorlie PD, Stafford RS, Turan TN, Turner MB, Wong ND, Wylie-Rosett J (2011). Heart disease and stroke statistics–2011 update: a report from the American Heart Association. Circulation.

[36] Bressler J, Folsom AR, Couper DJ, Volcik KA, Boerwinkle E (2010). Genetic variants identified in a European genome-wide association study that were found to predict incident coronary heart disease in the atherosclerosis risk in communities study. Am J Epidemiol.

[37] Hughes MF, Saarela O, Stritzke J, Kee F, Silander K, Klopp N, Kontto J, Karvanen J, Willenborg C, Salomaa V, Virtamo J, Amouyel P, Arveiler D, Ferrières J, Wiklund PG, Baumert J, Thorand B, Diemert P, Trégouët DA, Hengstenberg C, Peters A, Evans A, Koenig W, Erdmann J, Samani NJ, Kuulasmaa K, Schunkert H (2012). Genetic markers enhance coronary risk prediction in men: the MORGAM prospective cohorts. PLoS ONE.

[38] Sun YX, Gao CY, Lu Y, Fu X, Jia JG, Zhao YJ, Li LD, Dui HZ, Zhang XY, Li ZY, Lei L, Zhang WF, Yuan YQ (2017). Association between PPAP2B gene polymorphisms and coronary heart disease susceptibility in Chinese Han males and females. Oncotarget.

[39] Luo JY, Ma YT, Xie X, Yang YN, Li XM, Ma X, Yu Z, Chen BD, Liu F (2014). Association of intercellular adhesion molecule-1 gene polymorphism with coronary heart disease. Mol Med Rep.

[40] Ginter E, Simko V (2013). Women live longer than men. Bratisl Lek Listy.

[41] Joakimsen O, Bønaa KH, Stensland-Bugge E, Jacobsen BK (2000). Population-based study of age at menopause and ultrasound assessed carotid atherosclerosis: the Tromsø Study. J Clin Epidemiol.

[42] Çoban N, Onat A, Kömürcü Bayrak E, Güleç Ç, Can G, Erginel Ünaltuna N (2014). Gender specific association of ABCA1 gene R219K variant in coronary disease risk through interactions with serum triglyceride elevation in Turkish adults. Anadolu kardiyoloji dergisi: AKD = Anatol J Cardiol.

[43] Song J, Hao L, Wei W, Yang R, Wang C, Geng H, Li H, Wang S, Lu G, Feng T, Sun X, Liu S, Wang G, Cheng Y (2020). A SNP in the 3'UTR of the porcine IGF-1 gene interacts with miR-new14 to affect IGF-1 expression, proliferation and apoptosis of PK-15 cells. Domest Anim Endocrinol.

[44] Amini H, Shroff N, Stamova B, Ferino E, Carmona-Mora P, Zhan X, Sitorus PP, Hull H, Jickling GC, Sharp FR, Ander BP (2020). Genetic variation contributes to gene expression response in ischemic stroke: an eQTL study. Ann Clin Transl Neurol.

[45] Matana A, Ziros PG, Chartoumpekis DV, Renaud CO, Polašek O, Hayward C, Zemunik T, Sykiotis GP (2020). Rare and common genetic variations in the Keap1/Nrf2 antioxidant response pathway impact thyroglobulin gene expression and circulating levels, respectively. Biochem Pharmacol.

[46] Moore JH (2003). The ubiquitous nature of epistasis in determining susceptibility to common human diseases. Hum Hered.

[47] Saito Y, Tanaka A, Node K, Kobayashi Y (2021). Uric acid and cardiovascular disease: a clinical review. J Cardiol.

[48] Braga F, Pasqualetti S, Ferraro S, Panteghini M (2016). Hyperuricemia as risk factor for coronary heart disease incidence and mortality in the general population: a systematic review and meta-analysis. Clin Chem Lab Med.

[49] Dai XM, Wei L, Ma LL, Chen HY, Zhang ZJ, Ji ZF, Wu WL, Ma LY, Kong XF, Jiang LD (2015). Serum uric acid and its relationship with cardiovascular risk profile in Chinese patients with early-onset coronary artery disease. Clin Rheumatol.

[50] Yu W, Cheng JD (2020). Uric acid and cardiovascular disease: an update from molecular mechanism to clinical perspective. Front Pharmacol.

[51] Purnima S, El-Aal BG (2016). Serum uric acid as prognostic marker of coronary heart disease (CHD). Clinica e Investigacion en Arteriosclerosis: Publicacion Oficial de la Sociedad Espanola de Arteriosclerosis.

[52] Xie X, Ma YT, Yang YN, Li XM, Fu ZY, Zheng YY, Ma X, Chen BD, Liu F, Huang Y, Yu ZX, Chen Y (2012). Serum uric acid levels are associated with polymorphism in the SAA1 gene in Chinese subjects. PLoS ONE.

[53] Sun X, Jiang F, Zhang R, Tang SS, Chen M, Peng DF, Yan J, Wang T, Wang SY, Bao YQ, Hu C, Jia WP (2014). Serum uric acid levels are associated with polymorphisms in the SLC2A9, SF1, and GCKR genes in a Chinese population. Acta Pharmacol Sin.

[54] Wang C, Yan W, Wang H, Zhu J, Chen H (2019). APOE polymorphism is associated with blood lipid and serum uric acid metabolism in hypertension or coronary heart disease in a Chinese population. Pharmacogenomics.

