# Supplementary material for: The effect of CYP7B1 polymorphisms on the risk of coronary heart disease in Hainan Han population
Source: BMC Med Genomics. 2021 Sep 7;14:220. doi: 10.1186/s12920-021-01067-x (PMC8422734; doi:10.1186/s12920-021-01067-x)
Supplement: Supplementary file 1 — Additional file 1. Table S1 False-positive report probability analysis for the positive findings between CYP7B1 polymorphisms and CHD risk. [file 12920_2021_1067_MOESM1_ESM.docx]

Table S1 False-positive report probability analysis for the positive findings between *CYP7B1* polymorphisms and CHD risk

| Genotype and Variables | OR (95 % CI) | *p* Value ^a^ | Statistical Power ^b^ | Prior Probability | | | |  |
| --- | --- | --- | --- | --- | --- | --- | --- | --- |
|  |  |  |  | 0.25 | 0.1 | 0.01 | 0.001 | 0.0001 |
| **Age> 60 years** |  |  |  |  |  |  |  |  |
| rs6472155 *A* > *G* |  |  |  |  |  |  |  |  |
| *G* Vs *A* | 1.43 (1.10-1.86) | 0.008 | 0.994 | 0.023 ^c^ | 0.065 ^c^ | 0.433 | 0.885 | 0.987 |
| *GG* Vs *AA* | 2.20 (1.07-4.49) | 0.031 | 0.397 | 0.186 ^c^ | 0.407 | 0.883 | 0.987 | 0.999 |
| **Women** |  |  |  |  |  |  |  |  |
| rs6472155 *A* > *G* |  |  |  |  |  |  |  |  |
| *G* Vs *A* | 1.48 (1.06-2.07) | 0.022 | 0.961 | 0.064 ^c^ | 0.171 ^c^ | 0.694 | 0.958 | 0.996 |
| *GG* Vs *AA* | 3.17 (1.19-8.44) | 0.021 | 0.256 | 0.160 ^c^ | 0.514 | 0.921 | 0.992 | 0.999 |
| *GG* Vs *AA*-*AG* | 2.91 (1.12-7.58) | 0.029 | 0.221 | 0.180 ^c^ | 0.539 | 0.928 | 0.992 | 0.999 |
| **Drinking** |  |  |  |  |  |  |  |  |
| rs2980003 *C* > *T* |  |  |  |  |  |  |  |  |
| *T* Vs *C* | 0.57 (0.35-0.95) | 0.031 | 0.692 | 0.118 ^c^ | 0.287 | 0.816 | 0.978 | 0.998 |
| *TC* Vs *CC* | 0.48 (0.23-0.97) | 0.042 | 0.455 | 0.112 ^c^ | 0.447 | 0.899 | 0.989 | 0.999 |
| *TC*-*TT* Vs *CC* | 0.47 (0.24-0.91) | 0.025 | 0.427 | 0.150 ^c^ | 0.346 | 0.853 | 0.983 | 0.998 |
| **Non-drinking** |  |  |  |  |  |  |  |  |
| rs6472155 *A* > *G* |  |  |  |  |  |  |  |  |
| *GG* Vs *AA* | 3.16 (1.05-9.48) | 0.040 | 0.207 | 0.198 ^c^ | 0.635 | 0.950 | 0.995 | 0.998 |
| *GG* Vs *AA*-*AG* | 3.43 (1.16-10.09) | 0.025 | 0.164 | 0.199 ^c^ | 0.581 | 0.938 | 0.994 | 0.999 |

*p* value ^a^ was calculated by unconditional logistic regression analysis with adjustment for age and gender.

Statistical power ^b^ was calculated using the number of observations in the subgroup and the OR and *p* values in this table.

^c^ The level of false-positive report probability threshold was set at 0.2 and noteworthy findings are presented.
